# Letter to the editor of Heliyon re: Mapping sustainability reporting research with the UN's sustainable development goal. Heliyon 9 (2023) e18510

**DOI:** 10.1016/j.heliyon.2024.e34292

**Published:** 2024-07-09

**Authors:** Bakthavachalam Elango

**Affiliations:** a Department of Library and Information Science, Rajagiri College of Social Sciences, Kochi, 683 104, Kerala, India; b Centre of Excellence in Journalology, Rajagiri College of Social Sciences, Kochi, 683 104, Kerala, India

**Keywords:** Bibliometrics, CAGR, Compound Annual Growth Rate, Calculation Error

## Abstract

This letter to the editor discusses an investigation on sustainability reporting research in line with the UN's Sustainable Development Goals (SDGs). There was an error found in the Compound Annual Growth Rate (CAGR) calculation in the original research, which was rectified by applying the correct formula. Despite this error, the study's outcomes remained unaffected. It concludes by emphasizing the importance of a systematic approach in academic writing to learn from errors and avoid similar mistakes in the future.

## 1 Introduction

A recent investigation [1] examined the landscape of sustainability reporting research in relation to the Sustainable Development Goals set by the United Nations. Using bibliometric and science mapping techniques, the study examined patterns in research related to sustainability reporting based on the Scopus database and highlighted a significant increase in publications and citations after 2015. This increase is consistent with the increasing recognition of the SDGs. In addition, the research identified several key themes in sustainability reporting research, such as stakeholder engagement, corporate governance, sustainable development objectives, climate change, and global reporting initiatives. Finally, the study used social network analysis to examine the interconnectedness of the SDGs as presented in sustainability reporting literature and highlighted the importance of SDGs 9 and 12 as key themes.

## 2 Discussion

The authors record an increase in publications on sustainability reporting, which shows a steady increase from 107 in 2012 to 766 in 2022 and the number of citations has been increased from 57 in 2012 to 21419 in 2022 [1]. To take this development into account, the authors calculated the average Compound Annual Growth Rate (CAGR) at 14.67 % for the total number of publications and 29.69 % for the total number of citations (see page 7). An evaluation of the CAGR value revealed an error in the calculation process. Since the authors did not provide the CAGR formula, we assume that they used the following formula (Elango 2019) [2]:

(1) $$CAGR=\left({\left(\frac{V_{final}}{V_{begin}}\right)}^{\frac{1}{t-1}}-1\right)*100$$

where, t is the number of years.

By substituting the respective values into Formula 1 above, the CAGR is calculated to be 21.75 % for total publications and 80.92 % for total citations. After reviewing these calculated values, it appears that the numbers presented by the authors may contain inaccuracies. To authenticate the calculated value, the methodology advocated by Elango (2019: 2024) [2,3] is used in this correspondence. An analysis of the results (Table 1) revealed that the authors used an incorrect formula that led to inaccuracies, while the exact formula 1 corrects this error. For example, the number of publications should increase from 107 in 2012 to 766 in 2022. Using the authors' CAGR value (14.67 %), the projected increase is 421, resulting in a shortfall of 345. The number of citations should increase from 57 in 2012 to 21,419 in 2022. The authors' CAGR value (29.69 %) implies a potential increase to 767, resulting in a deficit of 20,652. Compared to the actual number of citations in 2022, the estimated value based on the authors' CAGR value appears to be significantly lower. This allows everyone to verify that the calculated CAGR is error-free.

**Table 1:** Comparison of growth of publications and citations.

Year	Total Publications		Total Citations	
	Growth by authors' CAGR	Growth by CAGR (1)	Growth by authors' CAGR	Growth by CAGR (1)
2012	107	107	57	57
2013	123	130	74	103
2014	141	159	96	187
2015	161	193	124	338
2016	185	235	161	611
2017	212	286	209	1105
2018	243	349	271	1999
2019	279	424	352	3617
2020	320	517	456	6544
2021	367	629	592	11839
2022	421	766	767	21419
Rounded-off				

## 3 Conclusion

Despite the presence of an error in the CAGR calculation, the study's results remained unaffected. Academic writing requires that researchers demonstrate a thorough and systematic approach to gain valuable insight from the errors highlighted in the conclusion and to use this understanding to prevent similar errors in future cases. Furthermore, it is important to emphasize that this communication focuses on the identification and analysis of errors rather than on individual researchers.

## Funding

None.

## Data availability statement

This manuscript includes all the information required to support the conclusions.

## CRediT authorship contribution statement

**Bakthavachalam Elango:** Conceptualization, Formal analysis, Investigation, Writing – original draft, Writing – review & editing.

## Declaration of competing interest

The authors declare that they have no known competing financial interests or personal relationships that could have appeared to influence the work reported in this paper.

## References

[1] Raman R., Nair V.K., Shivdas A., Bhukya R., Viswanathan P.K., Subramaniam N., Nedungadi P. (2023). Mapping sustainability reporting research with the UN's sustainable development goal. Heliyon.

[2] Elango B. (2019). Calculation errors in bibliometrics: the case of CAGR. COLLNET J. Sci. Inf. Manag..

[3] Elango B. (2024). G-20 countries and research collaboration. Curr. Sci..

